# Which Is More Useful in Predicting Hospital Mortality -Dichotomised Blood Test Results or Actual Test Values? A Retrospective Study in Two Hospitals

**DOI:** 10.1371/journal.pone.0046860

**Published:** 2012-10-15

**Authors:** Mohammed A. Mohammed, Gavin Rudge, Gordon Wood, Gary Smith, Vishal Nangalia, David Prytherch, Roger Holder, Jim Briggs

**Affiliations:** 1 Primary Care Clinical Sciences, University of Birmingham, Edgbaston, Birmingham, West Midlands, United Kingdom; 2 Public Health, University of Birmingham, Edgbaston, Birmingham, United Kingdom; 3 George Eliot Hospital, Nuneaton, Warwickshire, West Midlands, United Kingdom; 4 Centre of Postgraduate Medical Research & Education, The School of Health & Social Care, Bournemouth University, Bournemouth, Dorset, United Kingdom; 5 Centre for Anaesthesia, University College London Hospitals, London, United Kingdom; 6 Centre for Healthcare Modelling and Informatics, School of Computing, University of Portsmouth, Portsmouth, United Kingdom; Robert Wood Johnson Medical School, United States of America

## Abstract

**Background:**

Routine blood tests are an integral part of clinical medicine and in interpreting blood test results clinicians have two broad options. (1) Dichotomise the blood tests into normal/abnormal or (2) use the actual values and overlook the reference values. We refer to these as the “binary” and the “non-binary” strategy respectively. We investigate which strategy is better at predicting the risk of death in hospital based on seven routinely undertaken blood tests (albumin, creatinine, haemoglobin, potassium, sodium, urea, and white blood cell count) using tree models to implement the two strategies.

**Methodology:**

A retrospective database study of emergency admissions to an acute hospital during April 2009 to March 2010, involving 10,050 emergency admissions with routine blood tests undertaken within 24 hours of admission. We compared the area under the Receiver Operating Characteristics (ROC) curve for predicting in-hospital mortality using the binary and non-binary strategy.

**Results:**

The mortality rate was 6.98% (701/10050). The mean predicted risk of death in those who died was significantly (p-value <0.0001) lower using the binary strategy (risk = 0.181 95%CI: 0.193 to 0.210) versus the non-binary strategy (risk = 0.222 95%CI: 0.194 to 0.251), representing a risk difference of 28.74 deaths in the deceased patients (n = 701). The binary strategy had a significantly (p-value <0.0001) lower area under the ROC curve of 0.832 (95% CI: 0.819 to 0.845) versus the non-binary strategy (0.853 95% CI: 0.840 to 0.867). Similar results were obtained using data from another hospital.

**Conclusions:**

Dichotomising routine blood test results is less accurate in predicting in-hospital mortality than using actual test values because it underestimates the risk of death in patients who died. Further research into the use of actual blood test values in clinical decision making is required especially as the infrastructure to implement this potentially promising strategy already exists in most hospitals.

## Introduction

Blood tests are an integral part of clinical medicine and are routinely undertaken during a patient's stay in hospital. Typically, routine blood tests consist of a core list of seven biochemical and haematological tests, (albumin, creatinine, potassium, sodium, urea, haemoglobin and white blood cell count) and, in the absence of contraindications and subject to consent, almost all patients admitted to hospital undergo these tests on admission. There is increasing evidence of the relationship of individual, or groups of, abnormal laboratory results and in-hospital mortality [1]–[9].

Blood test results are reported with actual values and their respective reference ranges; values outside of the reference range are flagged as abnormal. In considering the information from blood test results the clinician has, broadly speaking, two options. (1) Dichotomise the blood tests results into normal/abnormal using the reference ranges or (2) make use of the actual values without particular attention to the reference ranges. We refer to these as the “binary” and the “non-binary” strategy respectively. However, it is unclear which strategy is likely to be the most effective in assessing the risk of mortality of patients admitted to hospital and, at least for now, a controlled trial comparing the two strategies is a premature proposition. Using a using decision tree-based desktop exercise, we investigated whether the binary or non-binary approach is more accurate in predicting the risk of death following emergency admission to hospital.

## Methods

### Ethical Approval

Ethical clearance was sought from two sources. For use of GE Hospital data, the lead author (MAM) sought advice from chair of the Birmingham research ethics committee and was advised that formal ethical approval was not necessary as this constitutes an audit/service evaluation. For the Portsmouth Hospital data, DP, obtained ethical approval from the Isle of Wight, Portsmouth & South East Hampshire Research Ethics Committee (Reference No: 08/02/1394).

### Setting and data

The data originate from a medium-sized acute hospital in central England consisting of about 400 beds serving a catchment population of about 300,000. All spells following emergency admission within a financial year, (April 2009 to March 2010), were included. Using the hospital administration system, for each admission we obtained the following: patient's age, gender, admission date/time, discharge date/time and discharge status on (alive/dead). The following were excluded -patients aged less than 16 years of age, admissions to the maternity unit or any admissions with missing or invalid data. Using a pseudonymised, unique patient identifier we linked these data to the hospital laboratory computer system to determine the index blood test, (within a 24 hour window either side of the admission date/time), for each patient. We included tests prior to the admission date because it is not unusual for patients to have blood tests in the Accident and Emergency (A&E) department just before being formally admitted to the hospital. Blood tests outside this ±24 hour window were not regarded as index blood tests and were excluded. Patients who did not have a blood test were also excluded.

We considered the following seven blood tests:-albumin (g/L: reference range 35 to 50), creatinine (µmol/L: male reference range 65 to 105, female reference range 50 to 90), haemoglobin (g/dL: male reference range 13 to 17, female reference range 12 to 15), potassium (mmol/L: reference range 3.5 to 5.3), sodium (mmol/L: reference range 133 to 146), urea (mmol/L: reference range 2 to 8) and white blood cell count (10^9^ cells/L: reference range 4 to 11). These reference ranges were extracted from the reported blood test and represent the majority of records. However, there were a very small number of records where the reference ranges for creatinine or haemoglobin or white blood cell count differed from those reported above. These records were retained in the analyses without any modifications. For 88 admissions the reported reference ranges for haemoglobin (g/dL) were 13 to 16 (62 females) and 13 to 16 (25 males). For 85 admissions the upper reference range for white blood cell count was 13 (10^9^ cells/L) and for one admission it was 14(10^9^ cells/L). For one female the reported reference range for creatinine (µmol/L) was 65 to 105.

### Implementation of the binary and non-binary strategy

Our primary analysis involves the use of Classification and Regression Trees (CART), a statistical data mining technique for constructing decision trees by recursively splitting or partitioning patients into homogenous groups [10]. CART analysis has been used previously to support medical decision making [11]–[13] although its use is still somewhat novel. Tree models are intuitive to interpret because they have (a) a simple visual presentation which starts by identifying the most important predictor variables, (b) naturally incorporate interaction effects, (c) identify cut-offs for continuous covariates, (d) are distribution free and (e) can handle non-linear relationships. Some of these characteristics reflect human decision making processes and are not readily accommodated within a standard logistic regression framework. When first developed, CART analysis could lead to quite large tree models, but recent work has incorporated p-value based tree modelling, known as conditional trees, which yield smaller tree models whilst simultaneously controlling for multiple testing, (Bonferroni adjustment, based on p≤0.01). They are available in the *Party* Package [14] in *R* [15]. Our purpose in using conditional tree models is to implement the two strategies and thereby enable a fair comparison of the two strategies without seeking to develop a clinical prediction model [11].

### Accuracy of the binary and non-binary strategy

In assessing the tree models for each strategy we considered their discrimination and calibration characteristics [11]. Discrimination relates to how well the strategy can separate, (or discriminate between), those who died and those who did not. Calibration relates to the agreement between observed mortality and predicted risk.

Overall statistical performance of the two strategies was assessed using the scaled Brier score which incorporates both discrimination and calibration. The Brier score is the squared difference between actual outcomes and predicted risk of death, scaled by the maximum Brier score such that the scaled Brier score ranges from 0–100%. Higher values indicate superior models.

The concordance statistic (*c*-statistic) is a commonly used measure of discrimination. For a binary outcome, the c-statistic is the area under the Receiver Operating Characteristics, (ROC) [16], curve. The ROC curve is a plot of the sensitivity, (true positive rate), versus 1-specificty, (false positive rate), for consecutive predicted risks. The area under the ROC curve is interpreted as the probability that a deceased patient has a higher risk of death than a randomly chosen non-deceased patient. A c-statistic of 0.5 is no better than tossing a coin, whilst a perfect model has a c-statistic of 1. Thus the higher the c-statistic the better the strategy. In general, values less than 0.7 are considered to show poor discrimination, values of 0.7–0.8 can be described as reasonable, and values above 0.8 suggest good discrimination. The two ROC curves were formally testing using DeLong's test for two correlated ROC curves (with a p<0.05 set a priori for statistical significance), implemented in the *pROCR* [17] package in *R*. Box plots showing the risk of death for those discharged alive and dead are a simple way to visualise the discrimination [18] of each strategy. The difference in the mean predicted risk of death for those who were discharged alive and died is a measure of the discrimination slope. The higher the slope the better the discrimination. We compared the difference in the discrimination slopes of each strategy. We also computed the mean predicted risk of death for alive and deceased discharges and used the Wilcoxon singed rank test (because predicted risks were not normally distributed even after transformations) for paired data to formally test the statistical significance of any differences using the two strategies. We used the Tukey mean-difference plot [19] (also known as the Bland-Altman plot [20]) to assess agreement in the predicted risks between the two strategies. We used scatter plots to explore the relationship between crude mortality and blood test result (divided into sixths). All analyses were undertaken in *R* [15].

### Generalisability

To assess the generalisability of our findings, we repeated the above analyses using a similarly constructed pseudonymised data frame over three years (January 2006 to December 2008) from Portsmouth Hospitals NHS Trust based on the index blood test (using reference ranges used in that hospital) but without requiring the blood test to have been performed within ±24 hours of admission. We report the c-statistic and discrimination slopes, from the binary versus non-binary strategy using data from Portsmouth hospitals. Other results (eg trees, ROC curves, mean-difference plots) are not reported.

## Results

There were 10050 emergency admissions during the year April 2009 to March 2010 with a complete set of blood test results within 24 hours of admission, with a mean age of 62.45 years (SD 21.41). Of these, 13.2% (1329/10050) underwent surgery. There were more females, (53.9%, 5419/10050) and the in-hospital mortality was 6.98%, (701/10050). Except for the white blood cell count (correlation 0.02, p = 0.09) all of the other blood tests showed significant correlations with age (all p<0.0001: albumin −0.43, creatinine 0.23, haemoglobin −0.28, potassium 0.14, sodium −0.19, urea 0.40).

**Figure 1:**
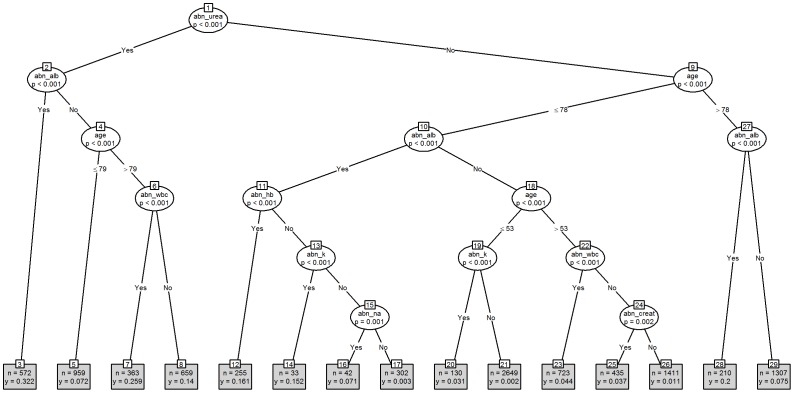
Binary Strategy as a tree model. Key: “abn_” prefix is abbreviation for Abnormal; alb = albumin; creat = creatinine; hb = haemoglobin; k = potassium; na = sodium; wbc = white blood cell count.

**Figure 2:**
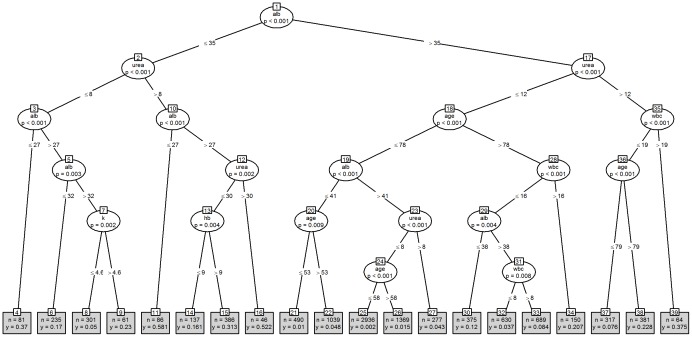
Non-binary strategy as a tree model. Key: Alb = albumin; hb = haemoglobin; k = potassium; na = sodium; wbc = white blood cell count.

Figure 1 shows the tree model based on the dichotomisation of the seven blood tests into normal/abnormal values, (the “binary” strategy), and Figure 2 shows the equivalent model using the actual blood test values, (the “non-binary” strategy). The binary strategy yields a tree with 29 nodes whilst the non-binary strategy yields a tree with 37 nodes, although the non-binary tree did not identify creatinine and sodium as significant risk factors for in-hospital mortality. Both trees identified significant interactions with age but gender was not seen in either tree. The binary tree begins with urea, whilst the non-binary tree begins with albumin. Under the binary strategy the lowest risk of death is associated with nodes 1∶9∶10∶18∶19∶21, (risk = 0.002, n = 2649). As examples, node 1 refers to the split between abnormal and normal blood urea results, whereas node 10 refers to the split between abnormal and normal blood albumin in samples with a normal blood urea result from patients aged 78 years or less. Interestingly, creatinine does not appear on this tree.

For the non-binary strategy the lowest risk of death is associated with nodes 1∶17∶18∶19∶23∶24∶25, (risk = 0.002, n = 2936). As examples, node 1 refers to the split at the level of an albumin above or below 35 g/L, whereas node 17 refers to a split at the level of a urea above or below 12 mmol/L in samples with an albumin above 35 g/L. Likewise, the highest risk of death under the binary strategy is associated with nodes 1∶2∶3, (risk = 0.322, n = 572), whilst for the non-binary strategy nodes 1∶2∶10, (risk = 0.581, n = 86) have the highest risk of death. Unlike the binary tree, creatinine does appear on this tree.

**Figure 3:**
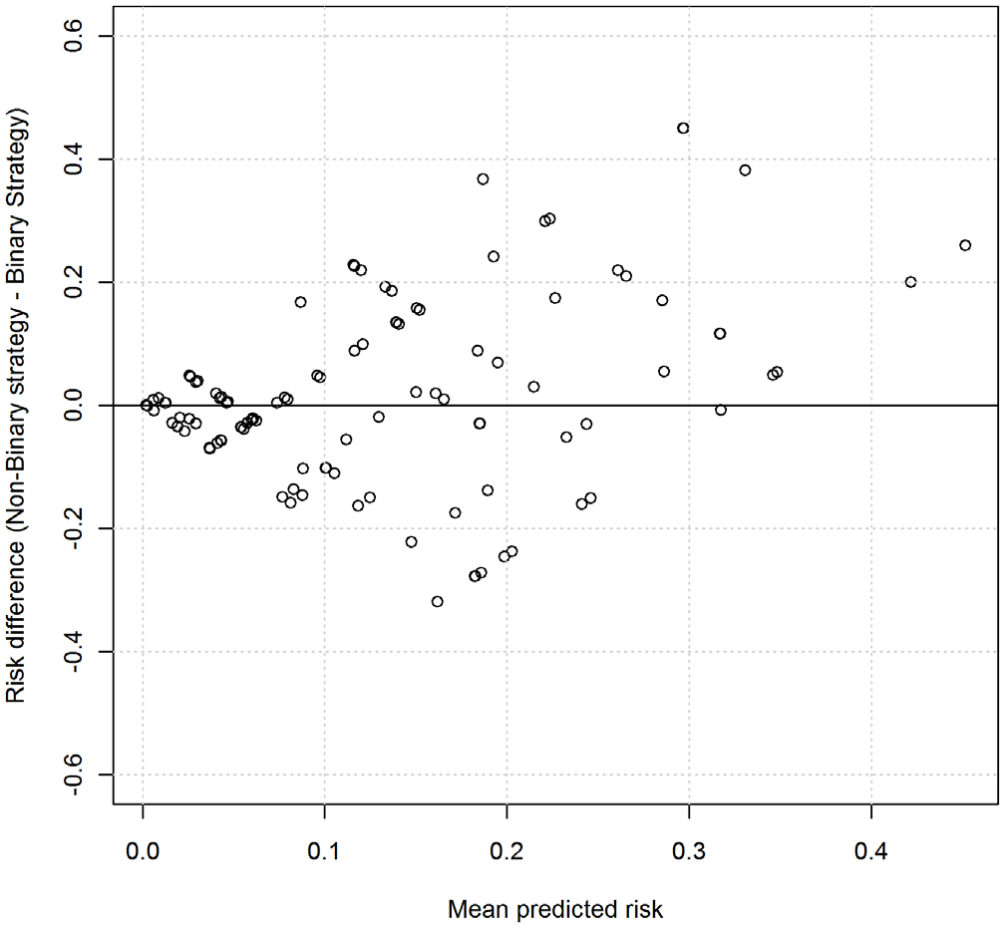
Mean-difference plot based on predicted risks of each strategy. Points are jittered with random noise to enhance visualisation.

**Figure 4:**
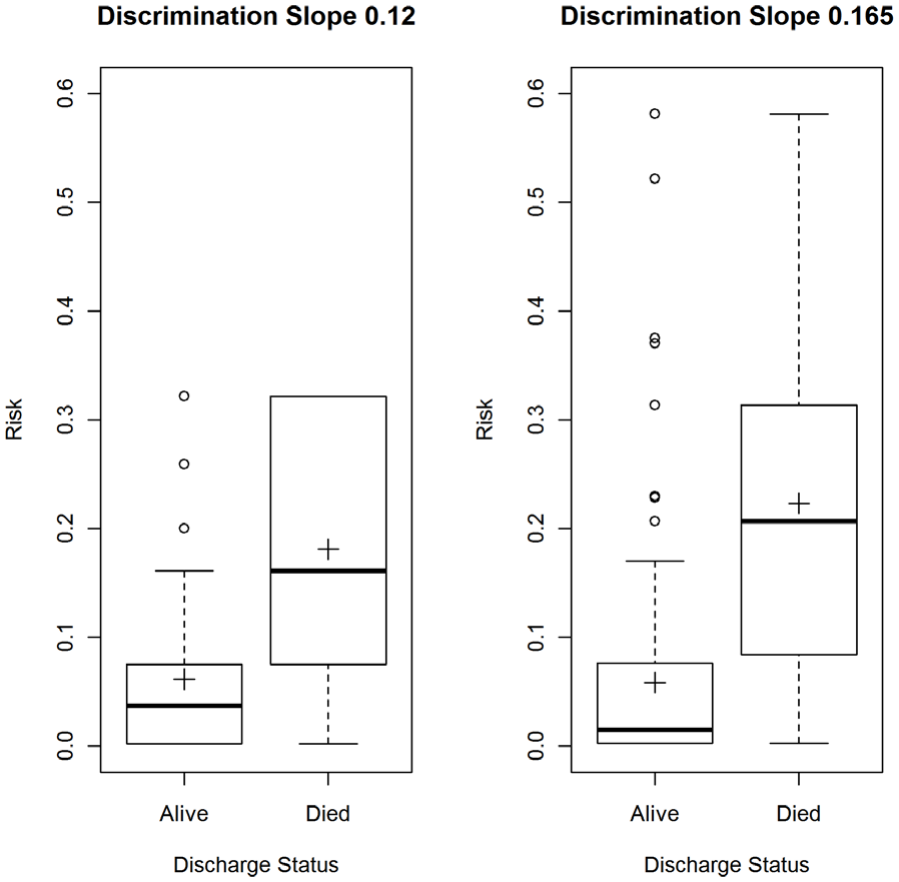
Box plots showing risk using the binary and non-binary strategy. Left panel is the binary strategy and right panel is the non-binary strategy. Cross indicates the mean.

Figure 3 shows the mean-difference plot of predicted risks using the two strategies. There is clear evidence of the differences increasing with mean risk, demonstrating that the two strategies have systematic disagreements, with the non-binary strategy producing systematically higher predicted risks. This can also be seen in Figure 4, which shows box plots of the risk of mortality for admissions where the patient was discharged alive and dead. The mean predicted risk of death in those discharged alive using the binary strategy was higher (risk = 0.0614, 95%CI: 0.0565 to 0.0663) compared with the non-binary strategy, (0.0582, 95%CI: 0.0534 to 0.0631), but this was not statistically significant (Wilcoxon signed rank test for paired data, p-value = 0.81). The mean predicted risk of death in those who died was significantly lower (Wilcoxon signed rank test for paired data, p-value<0.0001), using the binary strategy (risk = 0.181, 95%CI: 0.193 to 0.210), compared with the non-binary strategy (risk = 0.222, 95%CI: 0.194 to 0.251), representing a risk difference of 28.74 deaths (n = 701).

**Figure 5:**
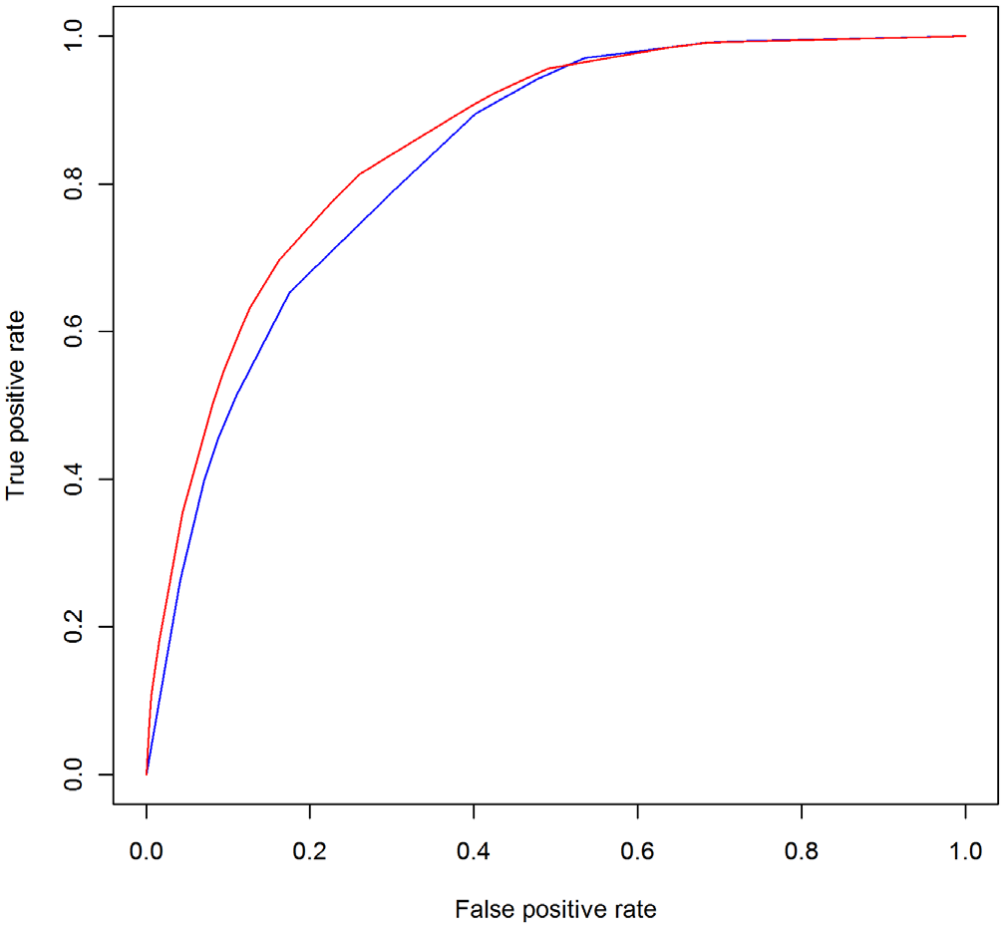
ROC curves for the binary and non-binary strategy. Blue is the binary strategy and red is the non-binary strategy.

Figure 5 shows the ROC curves with the binary strategy having a lower area under the ROC curve of 0.832 (95% CI: 0.819–0.845), compared to the non-binary strategy (0.853; 95% CI: 0.840 to 0.867 CI). This difference in the area under the two ROC curves was statistically significant, (DeLong's test for two correlated ROC curves, Z = 4.93, p-value<0.0001). The scaled Brier scores for the binary strategy were lower than the non-binary strategy (11.97% vs 16.42%).

**Figure 6:**
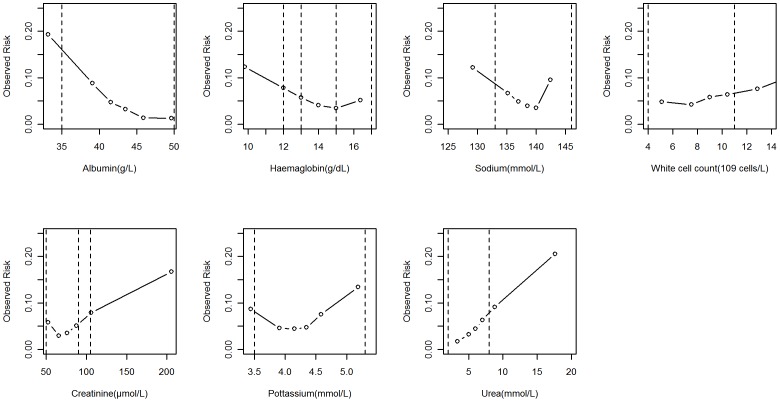
Observed mortality risk and blood test value. Dotted vertical lines are reference ranges). More than one pair of dotted lines indicates more than one pair of reference range, (eg for haemoglobin in men and women).

Figure 6 shows the relationship between a blood test result and crude mortality with reference ranges also indicated, (vertical lines). This figure shows why a binary interpretation of a blood test result is inadequate because it is an over simplification of predominantly non-linear relationships, even within the reference ranges.

### Generalisability

We used data from Portsmouth Hospitals NHS Trust to investigate the generalisability of our findings. There were 76964 emergency admissions during January 2006 to December 2008 with a complete set of index blood test results following admission. The mean age of admissions was 56.36 years (SD 25.68) with an in-hospital mortality of 5.41% (4160/76964). The mean predicted risk of death in those who died was significantly (p-value<0.0001) lower using the binary strategy (risk = 0.173 95%CI: 0.162 to 0.184) compared with the non-binary strategy (risk = 0.216 95%CI: 0.203 to 0.229), representing a risk difference of 178.88 deaths in the deceased patients (n = 4160). The binary strategy had a significantly (p-value<0.0001) lower area under the ROC curve of 0.866 (95% CI: 0.860 to 0.869) compared to the non-binary strategy (0.882 95% CI: 0878 to 0886 CI). The discrimination slope using the binary strategy was lower than under the non-binary strategy (0.126 vs 0.171). The scaled Brier scores for the binary strategy were lower than the non-binary strategy (12.57% vs 17.12%). These findings concur with those from GE Hospital.

## Discussion

The results of commonly measured biochemical and haematological tests are being increasing researched as potential predictors of a range of clinical outcomes. e.g., length of hospital stays, readmission and mortality [1]–[9]. Traditionally, whilst notice is often taken of specific test result values, many clinicians will initially decide upon the need for further investigation or treatment on the basis of whether the result value for a given substance, or group of substances, lies within the organisation's reference range. Using a large data set we have demonstrated that dichotomisation of routine blood test results is less accurate in predicting in-hospital mortality than using actual values, even those within the reference range, and that this is seen primarily in the under estimation of the risk of death in deceased patients. Our findings are consistent with the statistical axiom that dichotomisation, (or categorisation), is associated with loss of information and should be avoided [21], [22] and were replicated using data from another hospital.

However, perhaps the most important outcome of our study concerns the role of reference ranges in risk assessment following emergency admission. Reference ranges encompass ±1.96 standard deviations of the distribution of values from healthy individuals [23] whose underlying risk of mortality is by definition low. The non-binary strategy shows that it is possible, without any apparent detriment, to overlook the reference ranges when considering the risk of death. However, this does not suggest that reference ranges should be abandoned. Instead we should clarify that reference ranges provide a different perspective on the interpretation of a blood test result and that risk of a given outcome (eg death in our study) for all values within the reference range may not be constant.

In considering the binary and non-binary strategies we have implemented them using empirically derived decision trees, which are known to reflect some aspects of human decision making, (e.g. identifying cut-off values for continuous variables, accommodating interaction effects). Although in practice clinicians may adopt a combination of binary and non-binary strategies whilst taking account of the patient's medical history, vital signs and other relevant information above and beyond the blood tests results, the tree models used in our study are sophisticated implementations of these strategies with healthy concordance statistics. Nevertheless, our tree models should not be confused with the production and development of a clinical prediction model based on blood tests. Indeed, such a model has already been proposed [24]–[31], although the relatively low scaled Brier scores in our study caution against the exclusive use of these blood test results for predicting the risk of death following emergency admissions. Researchers have highlighted several advantages of using these seven blood test results to support clinical decision making and monitoring mortality in hospitals, which include the following:-(a) haematological and biochemical variables require only one venesection, (b) blood tests are undertaken as part of the process of care and unlike administrative databases [32] are not completed after the fact or susceptible to ‘gaming’, and (c) the information technology to enable real-time blood test based mortality risk assessment both within the laboratory and on the ward is already available in most hospitals. This latter point is important because whilst it might be possible for clinicians to implement the binary strategy without the aid of computers (although unlikely given evidence of human information processing limitations from cognitive psychology [33], [34]), the non-binary strategy would almost certainly require computer aided implementation. Indeed computer-aided implementation would allow more sophisticated approaches (eg Random Forests [35]) to be considered.

Our study reflects the use of index blood tests as part of the assessment of emergency admissions to a single hospital. To determine the extent to which our findings can be generalised to other uses of blood tests (e.g. chronic disease monitoring, to support differential diagnoses etc.) and the interpretation of consecutive blood tests requires further work. Nonetheless, our findings suggest that the non-binary strategy of interpreting blood test results may be superior in helping clinicians estimate the patient's risk of death and further studies aiming ultimately towards controlled trials are required, especially as the infrastructure to implement this potentially promising strategy already exists in most hospitals.

## Conclusion

Dichotomising routine blood test results is less accurate in predicting in-hospital mortality than using actual test values because it underestimates the risk of death in patients who died. Further research into the use of actual blood test values in clinical decision making is required especially as the infrastructure to implement this potentially promising strategy already exists in most hospitals.

## References

[1] Pine M, Jones B, Lou Y-B (1998) Laboratory values improve predictions of hospital mortality. Int J Qual Health Care 10: 491–501. 9928588 10.1093/intqhc/10.6.491

[2] Novack V, Pencina M, Zahger D, Fuchs L, Nevzorov R, et al (2010) Routine Laboratory Results and Thirty Day and One-Year Mortality Risk Following Hospitalization with Acute Decompensated Heart Failure. PLoS ONE 5: e12184 doi:10.1371/journal.pone.0012184. 20808904 10.1371/journal.pone.0012184 PMC2923147

[3] Vroonhof K, van Solinge WW, Rovers MM, Huisman A (2005) Differences in mortality on the basis of laboratory parameters in an unselected population at the Emergency Department. Clin Chem Lab Med 43: 536–41. 15899676 10.1515/CCLM.2005.093

[4] Asadollahi K, Beeching NJ, Gill G (2010) Leukocytosis as a predictor for non-infective mortality and morbidity. Q J Med 103: 285–292. 10.1093/qjmed/hcp182 20056764

[5] Asadollahi K, Beeching NJ, Gill G (2006) Hyponatraemia as a risk factor for hospital mortality. Q J Med 99: 877–880. 10.1093/qjmed/hcl120 17121769

[6] ten Boekel E, Vroonhof K, Huisman A, van Kampen C, de Kieviet W (2006) Clinical laboratory findings associated with in-hospital mortality. Clinica Chimica Acta 372: 1–13. 10.1016/j.cca.2006.03.024 16697361

[7] Asadollahi K, Beeching NJ, Gill G (2007) Hyperglycaemia and mortality. J R Soc Med 100: 503–507. 18048707 10.1258/jrsm.100.11.503 PMC2099401

[8] Evans NR, Dhatariya KK (2012) Assessing the relationship between admission glucose levels, subsequent length of hospital stay, readmission and mortality. Clinical Medicine Vol 12, No 2: 137–9. 10.7861/clinmedicine.12-2-137 PMC4954098 22586788

[9] Lan T-Y, Chiu H-C, Chang H-Y, Chang W-C, Chen H-Y, et al (2007) Clinical and laboratory predictors of all-cause mortality in older population. Arch Genrontol Geritatr 45: 327–334. 10.1016/j.archger.2007.02.001 17383026

[10] Breiman L, Friedman JH, Olshen R.A, Stone CJ (1984) Classification and regression trees. Monterey, CA: Wadsworth & Brooks/Cole Advanced Books & Software.

[11] Steyerberg EW (2009) Clinical Prediction Models. A practical approach to development, validation and updating. Springer.

[12] Harper PR (2005) A review and comparison of classification algorithms for medical decision making. Health Policy 71: 315–31. 15694499 10.1016/j.healthpol.2004.05.002

[13] Podgorelec V, Kokol P, Stiglic B, Rozman I (2002) Decision Trees: An Overview and Their Use in Medicine. J Med Syst 26 (5): 455–63. 10.1023/a:1016409317640 12182209

[14] Hothorn T, Hornik K, Zeileis A (2006) Unbiased Recursive Partitioning: A Conditional Inference Framework. J Comput Graph Stat 15 (3): 651–674.

[15] R Development Core Team (2011) R: A language and environment for statistical computing. R Foundation for Statistical Computing, Vienna, Austria. URL http://www.R-project.org. The R Project for Statistical Computing Accessed 2012 Sep 20.

[16] Hanley JA, McNeil BJ (1982) The meaning and use of the area under a receiver operating characteristic (ROC) curve. Radiology 143: 29–36. 7063747 10.1148/radiology.143.1.7063747

[17] Robin X, Turck N, Hainard A, Tiberti N, Lisacek F, et al pROC: an open-source package for R and S+ to analyze and compare ROC curves. BMC Bioinformatics 2011: 12–77 http://www.biomedcentral.com/14712105/12/77/. BioMed Central The Open Access Publisher Accessed 2012 Sep 20. 10.1186/1471-2105-12-77 PMC3068975 21414208

[18] Steyerberg EW, Vickers AJ, Cook NR, Gerds T, Gonen M, et al (2010) Assessing the Performance of Prediction Models. A Framework for Traditional and Novel Measures, Epidemiology 21: 128–138. 20010215 10.1097/EDE.0b013e3181c30fb2 PMC3575184

[19] Cleveland WS. Visualizing Data. (1993) Hobart Press, USA.

[20] Altman DG, Bland JM (1983) Measurement in medicine: the analysis of method comparison studies. The Statistician 32: 307–317.

[21] Altman D, Royston P (2006) The cost of dichotomising continuous variables. Statistics Notes. BMJ 332: 1080.1. 16675816 10.1136/bmj.332.7549.1080 PMC1458573

[22] Harell F (2001) Regression Modelling Strategies: with applications to linear models, logistic regression and survival analysis. Springer Series in Statistics.

[23] Bangert SK, Marshall WL (2008) Clinical biochemistry: metabolic and clinical aspects. Philadelphia: Churchill Livingstone/Elsevier. ISBN 0-443-10186-8.

[24] Prytherch DR, Sirl JS, Schmidt P, Featherstone PI, Weaver PC, et al (2005) The use of routine laboratory data to predict in-hospital morality in medical admissions. Resuscitation 66: 203–207. 15955609 10.1016/j.resuscitation.2005.02.011

[25] Prytherch DR, Sirl JS, Weaver PC, Schmidt P, Higgins B, et al (2003) Towards a national clinical minimum data set for general surgery. Br J Surg 90: 1300–5. 14515304 10.1002/bjs.4274

[26] Hucker TR, Mitchell GP, Blake LD, Cheek E, Bewick V, et al (2005) Identifying the sick: can biochemical measurements be used to aid decision making on presentation to the accident and emergency department. Br J Anaesth 94: 735–41. 15805142 10.1093/bja/aei122

[27] Asadollahi K, Hastings IM, Beeching NJ, Gill GV (2007) Laboratory risk factors for hospital mortality in acutely admitted patients. Q J Med 100: 501–507. 10.1093/qjmed/hcm055 17609227

[28] Asadollahi K, Hastings IM, Gill GV, Beeching NJ (2011) Prediction of hospital mortality from admission laboratory data and patient age: A simple model. Emergency Medicine Australasia 23: 354–363. 21668723 10.1111/j.1742-6723.2011.01410.x

[29] Pine M, Jones B, Lou YB (1998) Laboratory values improve predictions of hospital mortality. Int J Qual Health Care 10: 491–501. 9928588 10.1093/intqhc/10.6.491

[30] Vroonhof K, van Solinge WW, Rovers MM, Huisman A (2006) Differences in mortality on the basis of complete blood count in an unselected population at the emergency department. Lab Hematol 12: 134–8. 16950673

[31] Froom P, Shimoni Z (2006) Prediction of Hospital Mortality Rates by Admission Laboratory Tests. Clinical Chemistry 52: 325–8. 16449218 10.1373/clinchem.2005.059030

[32] Mohammed MA, Stevens AJ (2007) The value of administrative databases. BMJ 334: 1014–5 doi:10.1136/bmj.39211.453275.80. 17510106 10.1136/bmj.39211.453275.80 PMC1871738

[33] Sweller J (1988) Cognitive load during problem solving: Effects on learning. Cognitive Science 12: 257–285.

[34] van Merrienboer JJG, Sweller J (2010) Cognitive load theory in health professional education: design principles and strategies. Medical Education 44: 85–93. 20078759 10.1111/j.1365-2923.2009.03498.x

[35] Breiman L (2001) Random forests. Machine Learning 45 (1): 5–32.

